# Supplementary material for: Altered microRNA Transcriptome in Cultured Human Liver Cells upon Infection with Ebola Virus
Source: Int J Mol Sci. 2021 Apr 6;22(7):3792. doi: 10.3390/ijms22073792 (PMC8038836; doi:10.3390/ijms22073792)
Supplement: Supplementary file 1 [file ijms-22-03792-s001.zip › Supplementary_File/C_ GO_Analysis_Results/16-30nt_go_Makona-96h-Huh7_vs_Control-96h-Huh7_up.mature_mirna_targets/MF_Pvalue_tree.pdf]

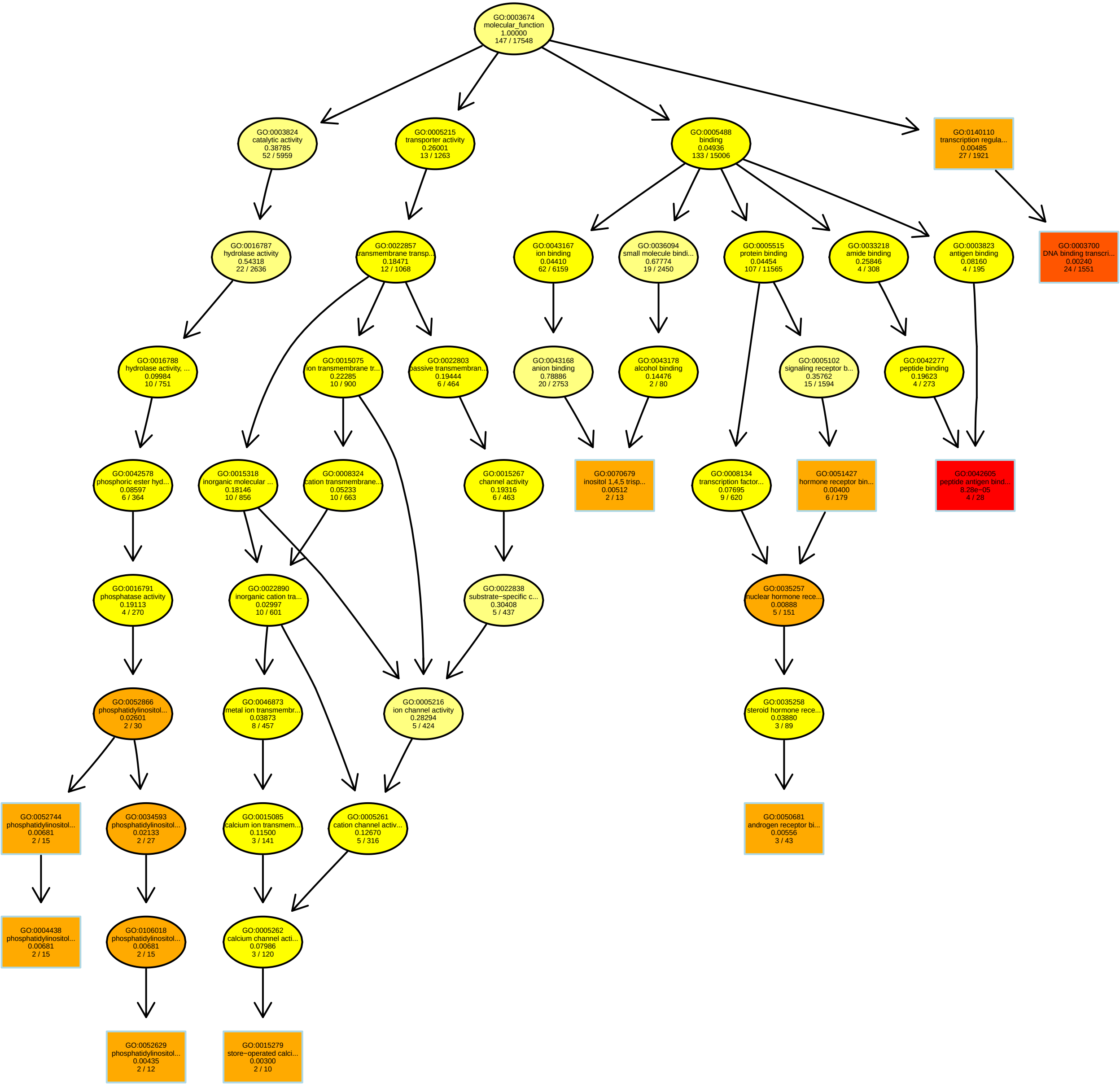
GO:0003674 molecular_function 1.00000 147 / 17548
GO:0003824 catalytic activity 0.38785 52 / 5959
GO:0005215 transporter activity 0.26001 13 / 1263
GO:0005488 binding 0.04936 133 / 15006
GO:0140110 transcription regula... 0.00485 27 / 1921
GO:0016787 hydrolase activity 0.54318 22 / 2636
GO:0022857 transmembrane transp... 0.18471 12 / 1068
GO:0043167 ion binding 0.04410 62 / 6159
GO:0036094 small molecule bindi... 0.67774 19 / 2450
GO:0005515 protein binding 0.04454 107 / 11565
GO:0033218 amide binding 0.25846 4 / 308
GO:0003823 antigen binding 0.08160 4 / 195
GO:0003700 DNA binding transcri... 0.00240 24 / 1551
GO:0016788 hydrolase activity, ... 0.09984 10 / 751
GO:0015075 ion transmembrane tr... 0.22285 10 / 900
GO:0022803 passive transmembran... 0.19444 6 / 464
GO:0043168 anion binding 0.78886 20 / 2753
GO:0043178 alcohol binding 0.14476 2 / 80
GO:0005102 signaling receptor b... 0.35762 15 / 1594
GO:0042277 peptide binding 0.19623 4 / 273
GO:0042578 phosphoric ester hyd... 0.08597 6 / 364
GO:0015318 inorganic molecular ... 0.18146 10 / 856
GO:0008324 cation transmembrane... 0.05233 10 / 663
GO:0015267 channel activity 0.19316 6 / 463
GO:0070679 inositol 1,4,5 trisp... 0.00512 2 / 13
GO:0008134 transcription factor... 0.07695 9 / 620
GO:0051427 hormone receptor bin... 0.00400 6 / 179
GO:0042605 peptide antigen bind... 8.28e−05 4 / 28
GO:0016791 phosphatase activity 0.19113 4 / 270
GO:0022890 inorganic cation tra... 0.02997 10 / 601
GO:0022838 substrate−specific c... 0.30408 5 / 437
GO:0035257 nuclear hormone rece... 0.00888 5 / 151
GO:0052866 phosphatidylinositol... 0.02601 2 / 30
GO:0046873 metal ion transmembr... 0.03873 8 / 457
GO:0005216 ion channel activity 0.28294 5 / 424
GO:0035258 steroid hormone rece... 0.03880 3 / 89
GO:0052744 phosphatidylinositol... 0.00681 2 / 15
GO:0034593 phosphatidylinositol... 0.02133 2 / 27
GO:0015085 calcium ion transmem... 0.11500 3 / 141
GO:0005261 cation channel activ... 0.12670 5 / 316
GO:0050681 androgen receptor bi... 0.00556 3 / 43
GO:0004438 phosphatidylinositol... 0.00681 2 / 15
GO:0106018 phosphatidylinositol... 0.00681 2 / 15
GO:0005262 calcium channel acti... 0.07986 3 / 120
GO:0052629 phosphatidylinositol... 0.00435 2 / 12
GO:0015279 store−operated calci... 0.00300 2 / 10
